# Estimating the Broad-Sense Heritability of Early Growth of Cowpea

**DOI:** 10.1155/2009/984521

**Published:** 2009-06-07

**Authors:** Nicole W. Xu, Shizhong Xu, Jeff Ehlers

**Affiliations:** ^1^John W. North High School, 1550 Third Street, Riverside, CA 92507, USA; ^2^Department of Botany and Plant Sciences, University of California, Riverside, CA 92521, USA

## Abstract

Cowpea is an important tropical crop. It provides a large proportion of the food resource for the African human population and their livestock. The yield and quality of cowpea have been dramatically improved through traditional breeding strategies for the past few decades. However, reports of heritability estimates for early growth of cowpea are rare. We designed a simple experiment to estimate the broad-sense heritability of early growth. We randomly selected 15 cowpea varieties among a total of 5000 cowpea accessions maintained in the cowpea breeding facility at the University of California, Riverside to examine the genetic determination of early growth of cowpea (measured as the height at day five after seeding). The estimated broad-sense heritability on the individual plant basis is 0.2190. However, the corresponding estimate on the plant mean basis (average of four plants) is 0.5198, which is very high for a quantitative trait. The high heritability may explain why traditional breeding for cowpea growth is so effective. Since the design of experiment and method of data analysis are novel, this report can serve as an educational note for students in the area of quantitative genetics and plant breeding.

## 1. Introduction

Cowpea (*Vigna unguiculata*) is a member of the Phaseoleae tribe of the Leguminosae family. Members of the Phaseoleae include many of the economically important warm season grain and oilseed legumes such as soybean (*Glycine max*), common bean (*Phaseolus vulgaris*), and mung bean (*Vigna radiata*). Cowpea plays a critical role in the lives of millions of people in Africa and other parts of the developing world where it is a major source of dietary protein that nutritionally complements staple low-protein cereal and tuber crops and is a valuable and dependable commodity that produces income for farmers and traders [1]. Cowpea is a valuable component of farming systems in many areas because of its ability to restore soil fertility for succeeding cereal crops grown in rotation with it [2, 3]. Early maturing cowpea varieties can provide the first food from the current harvest sooner than any other crop (in as few as 55 days after planting), thereby shortening the “hungry period” that often occurs just prior to harvest of the current season's crop in farming communities in the developing world. 

Cowpea is a self-fertilizing species, and varieties normally consist of a single homozygous line. A simple method commonly used to estimate trait heritability in cowpea is to measure the phenotypic variance among F_2_ individuals developed from the cross between two inbred lines. The total phenotypic variance among the F_2_ consists of the genetic variance and the environmental variance. The environmental variance can be estimated by the average of the phenotypic variance among plants of the parental lines. The difference between the phenotypic variance of the F_2_ individuals and the environmental variance estimated from the parents is the genetic variance. The ratio of the genetic variance to the total phenotypic variance is the estimated heritability. This type of analysis has a narrow inference space because it highly depends on the genetic differences between the two particular inbred lines selected. Therefore, heritability estimated from the cross of two inbred lines cannot be generalized to other populations or line crosses.

A method to estimate the broad-sense heritability with a larger inference space and that does not require hybridization and population development steps can be conducted by analyzing multiple lines for trait performance using simple analysis of variance. This note reports the estimated broad-sense heritability for early growth rate in cowpea from a simple experiment involving a selected sample of cowpea germplasm. We report the entire dataset collected from this experiment and the results of data analysis. This method can be applied to all other self-pollinated crops. The method can also be used by educators as an example to teach students in the area of plant breeding.

## 2. Material and Methods

### 2.1. Material

Seeds of 15 cowpea varieties (Table 1) were randomly selected from a total of 5000 cowpea accessions maintained in the cowpea breeding facility at the University of California, Riverside. Four seeds from each variety were planted in an 8 × 8 × 8 cm plastic pot. The green house temperature was maintained at 35°C day and 25°C night temperature to provide optimal growing conditions. The plants were watered in a 24-hour interval. Five days after the seeds were planted, the plant heights were measured. 

As a control group, seeds from one variety, CB46, were planted in 15 pots, each pot containing 4 plants. These plants were situated in the same green house as the experimental group and maintained using exactly the same temperature and water condition. Plant heights at day five were measured also for the control group. The data were collected and stored in Excel spread sheets for analysis.

### 2.2. Data Analysis

The following linear model was used for the analysis of variance. Let ${\overset{̅}{Y}}_{i}$ be the average height of the *i*th variety for *i* = 1,…, 15 from the experimental group. The linear model for ${\overset{̅}{Y}}_{i}$ is

(1) $${\overset{̅}{Y}}_{i}=\mu +\alpha_{i}+{\overset{̅}{\varepsilon }}_{i},$$

where *μ* is the grand mean, *α*_*i*_ is the genetic effect of the *i*th variety expressed as deviation from the mean, and ${\overset{̅}{\varepsilon }}_{i}$ is the environmental error for the *i*th variety. Let *α*_*i*_ ~ i.i.d. *N*(0, *σ*_*α*_^2^) and ${\overset{̅}{\varepsilon }}_{i}\;\sim \;\text{i}.\text{i}.\text{d}.\;N(0,\sigma_{\overset{̅}{\varepsilon }}^{2})$, where i.i.d. means independently and identically distributed. The total phenotypic variance is

(2) $$\sigma_{\overset{̅}{Y}}^{2}=\sigma_{\alpha }^{2}+\sigma_{\overset{̅}{\varepsilon }}^{2},$$

where *σ*_*α*_^2^ is the genetic variance of early growth rate of cowpea among the 15 varieties, and $\sigma_{\overset{̅}{\varepsilon }}^{2}$ is the environmental variance. 

Let ${\overset{̅}{X}}_{i}$ be the average height of the *i*th pot from the control group. Because all plants of the control group come from the same cultivar (CB46), the linear model for ${\overset{̅}{X}}_{i}$ is simply

(3) $${\overset{̅}{X}}_{i}=\mu +{\overset{̅}{\varepsilon }}_{i},$$

where *μ* is the grand mean, and ${\overset{̅}{\varepsilon }}_{i}$ is the environmental error for the *i*th pot with an assumed i.i.d. $\;N(0,\sigma_{\overset{̅}{\varepsilon }}^{2})$ distribution. The total phenotypic variance among all the pots within CB46 is

(4) $$\sigma_{\overset{̅}{X}}^{2}=\sigma_{\overset{̅}{\varepsilon }}^{2}.$$

From the experimental group, we obtain the total phenotypic variance of early growth. From the control group, we obtain the environmental variance. The difference leads to an estimate of the genetic variance,

(5) $$\sigma_{\alpha }^{2}=\sigma_{\overset{̅}{Y}}^{2}-\sigma_{\overset{̅}{X}}^{2}.$$

Therefore, the estimated broad-sense heritability for early growth of cowpea is

(6) $${\hat{H}}_{\text{mean}}^{2}=\frac{{\hat{\sigma }}_{\alpha }^{2}}{{\hat{\sigma }}_{\overset{̅}{Y}}^{2}}=\frac{{\hat{\sigma }}_{\overset{̅}{Y}}^{2}-{\hat{\sigma }}_{\overset{̅}{X}}^{2}}{{\hat{\sigma }}_{\overset{̅}{Y}}^{2}},$$

where

(7) $$\begin{array}{cc} {\hat{\sigma }}_{\overset{̅}{Y}}^{2} & =\frac{1}{15-1}\overset{15}{\underset{i=1}{\sum }}{\left({\overset{̅}{Y}}_{i}-\overset{̅}{\overset{̅}{Y}}\right)}^{2}=\frac{1}{15-1}\left[\overset{15}{\underset{i=1}{\sum }}{\overset{̅}{Y}}_{i}^{2}-\frac{1}{15}{\left(\overset{15}{\underset{i=1}{\sum }}{\overset{̅}{Y}}_{i}\right)}^{2}\right],\\ {\hat{\sigma }}_{\overset{̅}{X}}^{2} & =\frac{1}{15-1}\overset{15}{\underset{i=1}{\sum }}{\left({\overset{̅}{X}}_{i}-\overset{̅}{\overset{̅}{X}}\right)}^{2}=\frac{1}{15-1}\left[\overset{15}{\underset{i=1}{\sum }}{\overset{̅}{X}}_{i}^{2}-\frac{1}{15}{\left(\overset{15}{\underset{i=1}{\sum }}{\overset{̅}{X}}_{i}\right)}^{2}\right]. \end{array}$$

The broad-sense heritability based on the average height is called the heritability on the mean basis. The broad-sense heritability on the individual plant basis can be obtained using the following equation:

(8) $${\hat{H}}^{2}=\frac{{\hat{\sigma }}_{\alpha }^{2}}{{\hat{\sigma }}_{\alpha }^{2}+{\hat{\sigma }}_{\varepsilon }^{2}}=\frac{{\hat{\sigma }}_{\overset{̅}{Y}}^{2}-{\hat{\sigma }}_{\overset{̅}{X}}^{2}}{{\hat{\sigma }}_{\overset{̅}{Y}}^{2}+\left(k_{0}-1\right){\hat{\sigma }}_{\overset{̅}{X}}^{2}}.$$

The reason for the above adjustment is due to the fact that ${\hat{\sigma }}_{\alpha }^{2}=\sigma_{\overset{̅}{Y}}^{2}-\sigma_{\overset{̅}{X}}^{2}$, and ${\hat{\sigma }}_{\varepsilon }^{2}=k_{0}{\hat{\sigma }}_{\overset{̅}{\varepsilon }}^{2}=k_{0}\sigma_{\overset{̅}{X}}^{2}$, which leads to

(9) $${\hat{\sigma }}_{\alpha }^{2}+{\hat{\sigma }}_{\varepsilon }^{2}={\hat{\sigma }}_{\overset{̅}{Y}}^{2}-{\hat{\sigma }}_{\overset{̅}{X}}^{2}+k_{0}{\hat{\sigma }}_{\overset{̅}{X}}^{2}={\hat{\sigma }}_{\overset{̅}{Y}}^{2}+\left(k_{0}-1\right){\hat{\sigma }}_{\overset{̅}{X}}^{2},$$

where *k*_0_ = 4 is the number of plants within each pot for the control group. For unbalanced data, that is, some pots may contain a less number of plants due to unexpected random error, *k*_0_ < 4 (see [4] for the calculation of *k*_0_).

## 3. Results

The individual plant heights and the average heights from the experimental groups are listed in Table 1 while the corresponding values from the control group are given in Table 2. The estimated variances of early growth are $\sigma_{\overset{̅}{Y}}^{2}=2.2803,$ and $\sigma_{\overset{̅}{X}}^{2}=1.0949,$ respectively, for the two groups. Therefore, the estimated broad-sense heritability on the mean basis is

(10) $${\hat{H}}_{\text{mean}}^{2}=\frac{2.2803-1.0949}{2.2803}=\frac{1.1854}{2.2803}=0.5198.$$

In the control group, two plants died accidently before the experiment ended, and they occurred in two different pots. This produced unbalanced data. The adjusted average plant number within each pot became *k*_0_ = 3.86 [4]. After adjusting for the mean, the broad-sense heritability on the individual plant basis is

(11) $${\hat{H}}_{\text{individual}}^{2}=\frac{2.2803-1.0949}{2.2803+\left(3.86-1\right)\times 1.0949}=\frac{1.1854}{5.4227}=0.2190.$$

The broad-sense heritability on the individual plant basis should be reported, which is 0.2190 for the early growth of cowpea. This heritability is relatively low. However, in cowpea breeding programs, breeders often choose to evaluate performance of all varieties on the pot (plot) mean basis because cowpea is a self-pollinated plant. Therefore, the plot mean-based broad-sense heritability is more useful. Note that the plot mean-based broad-sense heritability can reach up to 1 if the number of plants within each pot is infinity.

## 4. Discussion

This study demonstrates a simple method to estimate the broad-sense heritability for early growth in cowpea. The sample size (15 cultivars each replicated 4 times) is not necessarily large. We obtained the estimated broad-sense heritability on the individual plant basis of 0.2190. We also evaluated the precision of the estimate using the Delta approximation [4]. The standard error for this estimate is 0.1645, which is quite high due to the small sample size. Therefore, the estimated broad-sense heritability on the individual plant basis should be reported as

(12) $${\hat{H}}_{\text{individual}}^{2}=0.2190\pm 0.1645.$$

The corresponding estimate of the broad-sense heritability on the plot mean basis is

(13) $${\hat{H}}_{\text{mean}}^{2}=0.5198\pm 0.2401.$$

We now have two estimated heritabilities, the individual plant-based heritability and the plot mean-based heritability. Which one is the one used in plant breeding? This depends on whether plot means are used or individual plants are used to measure the growth rates of cowpea. In most plant breeding experiments, plant means are used, especially for self-pollinated crops. Therefore, the plant mean-based heritability is more often used than the individual plant based heritability in plant breeding. The individual plant based heritability can be used in marker-assisted selection or molecular breeding.

Dry grain for human consumption is the most important product of the cowpea plant, but fresh or dried leaves (in many parts of Asia and Africa) [5], fresh peas (the southeastern US and Senegal), and fresh green pods (“longbean” or “asparagus bean” humid regions of Asia and in the Caribbean) may be the most important in some local situations. Cowpea hay plays a particularly critical role in feeding animals during the dry season in many parts of West Africa [2, 6, 7]. Therefore, growth rate of cowpea itself is an important trait. The high estimated broad-sense heritability of the trait makes genetic improvement of fast growth easy through traditional breeding program and molecular marker-aided selection. 

We used (5) to estimate the genetic variance. The validity of this equation is based on an assumption that there is no genotype by environment (G×E) interaction because we used the environmental variance for a single cultivar CB46 to represent the overall environmental variance. In future studies, more cultivars should be selected to provide a more accurate estimate of the environmental variance. Due to the constraint of facility of the experiment, only 15 cultivars were selected out of 5000 existing cultivars. The sample size is quite small and thus the inference space may not cover the entire collection of the cultivars. In future studies, at least 30 cultivars should be randomly selected to prevent error caused by random genetic drift. One reviewer suggested that we should select the limited number of cultivars from those representing a particular region of Africa so that the result can be directly used by cowpea breeding in that region.

## Figures and Tables

**Table 1:** Early growth rates (height at day 5 measured in cm) of 15 randomly selected varieties of cowpea ($\bar{Y}$ data).

Variety	Origin	Plant 1	Plant 2	Plant 3	Plant 4	Average ($\overset{̅}{Y}$)
CB46	California	4.0	4.5	11.5	—	6.66
UCR779	Botswana	9.0	9.5	8.5	7.5	8.62
IT97K-499-39	Nigeria	7.0	9.5	10.5	9.0	9.00
IT95K-1479	Nigeria	6.5	8.5	7.5	7.0	7.37
24-125B-1	Cameroon	8.5	8.5	8.5	9.5	8.75
IT93K-503-1	Nigeria	10.0	7.5	8.5	11.0	9.25
IT93K-693-2	Nigeria	10.0	11.5	10.5	11.5	10.87
IT84S-2049	Nigeria	9.5	9.0	10.0	9.0	9.37
Prima	Nigeria	10.0	11.0	9.0	10.0	10.00
524B	California	9.5	10.0	9.0	10.5	9.75
IT97K-819-132	Nigeria	5.0	7.5	7.5	7.0	6.75
IT82E-18	Nigeria	10.0	9.5	10.0	9.5	9.75
Bambe21	Senegal	12.0	11.0	12.0	12.0	11.75
SuVita2	Burkina Faso	12.0	11.0	10.5	12.0	11.37
KVx61-1-1	Burkina Faso	8.5	9.0	8.5	—	8.66

**Table 2:** Early growth rates (height at day 5 measured in cm) of 15 randomly selected pots of cowpea from the same variety (CB46) (the data).

Variety	Pot	Plant 1	Plant 2	Plant 3	Plant 4	Average ($\overset{̅}{X}$)
CB46	R1	9.0	8.0	8.0	9.5	8.62
CB46	R2	12.5	12.5	11.0	12.5	12.12
CB46	R3	10.5	9.0	8.5	—	9.33
CB46	R4	9.5	10.5	9.5	7.0	9.12
CB46	R5	8.0	8.0	7.5	8.0	7.87
CB46	R6	8.5	10.0	8.5	8.5	8.87
CB46	R7	5.0	11.0	9.0	8.5	8.37
CB46	R8	9.5	9.0	8.5	8.5	8.87
CB46	R9	8.5	9.0	9.5	9.0	9.00
CB46	R10	8.0	7.0	6.5	7.5	7.25
CB46	R11	8.0	7.5	9.5	10.0	8.75
CB46	R12	8.5	7.5	9.0	9.0	8.50
CB46	R13	10.0	9.5	8.0	7.0	8.62
CB46	R14	7.0	9.5	9.0	—	8.50
CB46	R15	7.5	8.0	8.0	8.0	7.87
